# Association of Genetic Predisposition and Physical Activity With Risk of Gestational Diabetes in Nulliparous Women

**DOI:** 10.1001/jamanetworkopen.2022.29158

**Published:** 2022-08-30

**Authors:** Kymberleigh A. Pagel, Hoyin Chu, Rashika Ramola, Rafael F. Guerrero, Judith H. Chung, Samuel Parry, Uma M. Reddy, Robert M. Silver, Jonathan G. Steller, Lynn M. Yee, Ronald J. Wapner, Matthew W. Hahn, Sriraam Natarajan, David M. Haas, Predrag Radivojac

**Affiliations:** 1 Department of Computer Science, Indiana University, Bloomington; 2 Institute of Computational Medicine, Johns Hopkins University, Baltimore, Maryland; 3 Khoury College of Computer Sciences, Northeastern University, Boston, Massachusetts; 4 Dana-Farber Cancer Institute, Boston, Massachusetts; 5 Department of Biological Sciences, North Carolina State University, Raleigh; 6 Department of Obstetrics and Gynecology, University of California, Irvine; 7 Department of Obstetrics and Gynecology, University of Pennsylvania School of Medicine, Philadelphia; 8 Department of Obstetrics, Gynecology, and Reproductive Sciences, Yale School of Medicine, Yale University, New Haven, Connecticut; 9 Department of Obstetrics and Gynecology, University of Utah School of Medicine, Salt Lake City; 10 Department of Obstetrics and Gynecology, Northwestern University Feinberg School of Medicine, Chicago, Illinois; 11 College of Physicians and Surgeons, Columbia University, New York, New York; 12 Department of Biology, Indiana University, Bloomington; 13 Department of Computer Science, The University of Texas at Dallas; 14 Department of Obstetrics and Gynecology, Indiana University School of Medicine, Indianapolis

## Abstract

**Importance:**

Polygenic risk scores (PRS) for type 2 diabetes (T2D) can improve risk prediction for gestational diabetes (GD), yet the strength of the association between genetic and lifestyle risk factors has not been quantified.

**Objective:**

To assess the association of PRS and physical activity in existing GD risk models and identify patient subgroups who may receive the most benefits from a PRS or physical activity intervention.

**Design, Settings, and Participants:**

The Nulliparous Pregnancy Outcomes Study: Monitoring Mothers-to-Be cohort was established to study individuals without previous pregnancy lasting at least 20 weeks (nulliparous) and to elucidate factors associated with adverse pregnancy outcomes. A subcohort of 3533 participants with European ancestry was used for risk assessment and performance evaluation. Participants were enrolled from October 5, 2010, to December 3, 2013, and underwent genotyping between February 19, 2019, and February 28, 2020. Data were analyzed from September 15, 2020, to November 10, 2021.

**Exposures:**

Self-reported total physical activity in early pregnancy was quantified as metabolic equivalents of task (METs). Polygenic risk scores were calculated for T2D using contributions of 84 single nucleotide variants, weighted by their association in the Diabetes Genetics Replication and Meta-analysis Consortium data.

**Main Outcomes and Measures:**

Estimation of the development of GD from clinical, genetic, and environmental variables collected in early pregnancy, assessed using measures of model discrimination. Odds ratios and positive likelihood ratios were used to evaluate the association of PRS and physical activity with GD risk.

**Results:**

A total of 3533 women were included in this analysis (mean [SD] age, 28.6 [4.9] years). In high-risk population subgroups (body mass index ≥25 or aged ≥35 years), individuals with high PRS (top 25th percentile) or low activity levels (METs <450) had increased odds of a GD diagnosis of 25% to 75%. Compared with the general population, participants with both high PRS and low activity levels had higher odds of a GD diagnosis (odds ratio, 3.4 [95% CI, 2.3-5.3]), whereas participants with low PRS and high METs had significantly reduced risk of a GD diagnosis (odds ratio, 0.5 [95% CI, 0.3-0.9]; *P* = .01).

**Conclusions and Relevance:**

In this cohort study, the addition of PRS was associated with the stratified risk of GD diagnosis among high-risk patient subgroups, suggesting the benefits of targeted PRS ascertainment to encourage early intervention.

## Key Points

**Question:**

Are genetic predisposition to diabetes and physical activity in early pregnancy cooperatively associated with risk of gestational diabetes (GD) among nulliparous women?

**Findings:**

In this cohort study of 3533 women, a high polygenic risk score (PRS) and low level of physical activity were associated with increased risk of GD. The estimated odds for participants with high PRS and low level of physical activity was 3.4 but was near or less than the baseline level of 1.0 with either low PRS or high activity.

**Meaning:**

These findings suggest that physical activity in early pregnancy is associated with reduced risk of GD and reversal of excess risk in genetically predisposed individuals, and PRS may have utility in identifying women for targeted interventions.

## Teaser

This cohort study assesses the association of polygenic risk score and physical activity with risk of gestational diabetes among nulliparous women.

## Introduction

Every year, approximately 7% of pregnancies in the US are affected by gestational diabetes (GD),^1^ and the risk for developing type 2 diabetes (T2D) has doubled in the past decade among patients with GD.^2^ Gestational diabetes has been shown to increase the longer-term risk of maternal T2D, cardiovascular morbidity, and kidney disease, as well as to increase the risk for T2D, obesity, and neuropsychiatric morbidity in offspring.^3^ To reduce the incidence of GD and accompanying adverse perinatal outcomes, effective and targeted risk reduction strategies among high-risk individuals need to be implemented.

The strongest risk factors for the development of GD are adverse outcomes in previous pregnancies.^4,5^ However, this information is unavailable for 40% of all pregnancies in the US that occur in nulliparous women.^6^ Additional characteristics associated with elevated risk of GD include obesity, low levels of physical activity, 35 years or older, and family history of diabetes.^7,8,9,10,11^ Despite these insights, the estimation of adverse perinatal outcomes in first pregnancies has remained challenging, and the search for additional GD risk factors applicable to nulliparous women remains ongoing.^12^

Significant progress has been made in identifying genetic variants associated with T2D and GD.^13^ Studies incorporating polygenic risk scores (PRS) generated using loci associated with T2D showed modest improvement in performance for GD prediction models when compared with a baseline clinical model.^14^ However, the added value of physical activity to these models has not been assessed. Moreover, the association of PRS and physical activity with the incidence of GD, and their association with other risk factors, also remains understudied. Herein, using the Nulliparous Pregnancy Outcomes Study: Monitoring Mothers-to-Be (nuMoM2b) observational cohort study of nulliparous women,^15^ we provide evidence of the association of exercise and PRS—both individually and jointly—with the risk of GD.

## Methods

### Study Population

This study population included individuals who were enrolled in an observational nuMoM2b cohort study in which nulliparous women were recruited from hospitals affiliated with 8 clinical sites in the US.^15^ Each site’s local governing institutional review board approved the study and all participants provided written informed consent before participation. Participants were enrolled from October 5, 2010, to December 3, 2013, and 4 study visits were scheduled at 6 to 13, 16 to 21, and 22 to 29 weeks of gestation and at the time of the delivery.

The study population for this work was selected from the original nuMoM2b participants as follows. Individuals missing all covariates (n = 10), those with prediabetes (n = 151), those with a diagnosis of or treatment for diabetes before pregnancy (n = 42), and those who did not undergo testing for GD or who had low-quality genotyping results (n = 467) were excluded from the analysis. Of the remaining 9368 participants, those who did not self-report White race (n = 3702), whose ancestry was not inferred to be European using SNPweights, version 2.1 (Program in Genetic Epidemiology and Statistical Genetics, Harvard T.H. Chan School of Public Health)^16^ (n = 632), and with incomplete or erroneous physical activity data (n = 1214) or PRS data (n = 287) were excluded. The remaining 3533 participants (132 with GD [3.7%]) were included for further analysis (eFigure 1 in the Supplement). Our study followed the Strengthening the Reporting of Observational Studies in Epidemiology (STROBE) reporting guideline.

### Covariates

Data were collected for the nuMoM2b cohort through interviews, self-administered questionnaires, clinical measurements, and medical records. Pregnancy outcome information was collected from medical records, and maternal blood samples were collected for DNA.^17^ Information on leisure physical activities during pregnancy was reported at study visits 1 to 3 using questions adapted from the Behavior Risk Factor Surveillance System.^18^ Participants’ physical activity during the previous 4 weeks was reported as the number of times per week and duration in minutes for their most frequently performed activities. The activities were translated to total metabolic equivalents of task (METs), calculated as the weighted sum of the minutes spent in each physical activity.^19^ Consistent with the Nurses’ Health Study II,^20^ participants were categorized as less active (n = 1168) and more active (n = 2365) based on a METs threshold of 450, which is equivalent to 150 minutes of moderate physical activity or 75 minutes of vigorous physical activity per week. All time-varying covariates used in this work, including body mass index (BMI; calculated as weight in kilograms divided by height in meters squared), waist circumference, and physical activity data, pertain to the first study visit.

### Genotypes

Genetic data were obtained from Guerrero et al,^16^ who recently reported genotypes for 97% of the nuMoM2b participants (n = 9757). Genotyping was performed between February 19, 2019, and February 28, 2020, using a commercially available kit (Infinium Multi-Ethnic Global D2 BeadChip; Illumina), yielding calls for 1 748 280 loci.^16^ We filtered the genotype set for quality control, excluding single nucleotide variants (SNVs) with missingness of greater than 0.02, minor allele frequency less than 0.01, Hardy-Weinberg equilibrium *P* < 1 × 10^−6^, and individuals with a call rate less than 98% or relatedness *F*_het_ greater than 0.2. We then used the filtered genotypes to generate a larger imputed data set, with a prephasing and imputation stepwise approach implemented in Eagle and Minimac3 (variable chunk size of 132 genomic chunks and default parameters), against the 1000 Genomes Project phase 3 reference.^21^ We only retained imputed SNVs for which the probability of genotype assignment was greater than 80%, with an INFO score greater than 0.1 and minor allele frequency greater than 0.005.

### Calculation of Genetic Risk

Genetic risk was estimated by applying an existing PRS for T2D derived from the Diabetes Genetics Replication and Meta-analysis Consortium data,^22^ following the methods of Powe et al.^23^ We limited our PRS calculations to individuals of European genetic ancestry for whom the T2D PRS was developed. An individual’s PRS was the sum of the number of risk alleles carried, each weighted by their corresponding β coefficient (estimated by Powe et al^23^). The calculated scores include contributions of 84 SNVs (of 85 included by Powe et al^23^) with high-quality data (all INFO scores >0.45; mean INFO score, 0.94; 37 SNVs were genotyped directly).

### Primary Outcome

The presence of GD was determined based on one of the following glucose tolerance testing (GTT) criteria: (1) fasting 3-hour 100-g GTT with 2 abnormal values among the following: fasting level at least 95 mg/dL, 1-hour level at least 180 mg/dL, 2-hour level at least 155 mg/dL, and 3-hour level at least 140 mg/dL; (2) fasting 2-hour 75-g GTT with 1 abnormal value among the following: fasting level at least 92 mg/dL, 1-hour level at least 180 mg/dL, or 2-hour level at least 153 mg/dL; and (3) nonfasting 50-g GTT level at least 200 mg/dL if no fasting 3-hour or 2-hour GTT was performed (to convert mg/dL to mmol/L, multiply by 0.0555). It was additionally noted whether GD was diagnosed during clinical care. In the absence of GTT data, clinical diagnosis was used to determine GD. All outcomes were collected by certified medical record abstractors at each clinical site to determine GD diagnosis.^15^

### Models and Evaluation

We developed a baseline model using features taken from screening questions by Artzi et al.^4^ The features include age, BMI, race and ethnicity, family history of diabetes, polycystic ovary syndrome diagnosis, high blood pressure diagnosis, diabetes diagnosis, history of GD, and previous measurement of hemoglobin A_1c_ level. Adapting the baseline model to features available to nulliparous women in the nuMoM2b cohort, we omitted pregnancy history and hemoglobin A_1c_ level from the feature list and incorporated waist size as an additional feature. The performance levels of classification models were measured using areas under the receiving operating characteristic curve (AUCs),^24^ estimated via 10-fold cross-validation. The 95% CIs were determined using 100 bootstrapping iterations.

The test for interaction between covariates was performed using the following model:

logit(*P*(*Y* = 1| *X*_1_, *X*_2_, *X*_3_, *X*_4_)) = *a* + *b X*_1_ + *c X*_2_ + *d X*_3_ + *e X*_4_ + *f X*_3_
*X*_4_ + ε

where *Y* is a binary response representing GD diagnosis (*Y* = 1 indicates presence of GD); *X*_1_ and *X*_2_ are age and BMI continuous random variables, respectively, used as confounders; *X*_3_ and *X*_4_ are binary random variables representing high PRS and low METs, respectively; ε is a zero-mean gaussian random variable with unknown variance σ^2^; and (*a*, *b*, *c*, *d*, *e*, *f*) is a set of real-valued parameters that, along with σ^2^, were estimated from data. The *P* value for coefficient *f* (null hypothesis: *f* = 0) was used to demonstrate a nonadditive association between *X*_3_ and *X*_4_, that is, high PRS and low METs.

### Statistical Analysis

Data were analyzed from September 15, 2020, to November 10, 2021. Unless otherwise stated, the odds ratio (OR)^25^ of each subgroup was calculated using the rest of the cohort who were not in the considered subgroup, and *P* values were obtained using the Fisher exact test. Positive likelihood ratio (LR)^25^ values were calculated as the ratio of posterior to prior odds of a GD diagnosis, where the prior odds refer to the odds of a GD diagnosis in a parent group and the posterior odds refer to the odds of a GD diagnosis in a subgroup of the parent group (ie, the parent group with additional criteria imposed). Positive LR *P* values were obtained by calculating the proportion of samples in which the child subgroup had a lower (or higher, as appropriate) LR than the parent subgroup using 10 000 bootstrapped samples of the analysis cohort.^26^ Data preprocessing was performed using MATLAB, release 2021a (MathWorks). Statistical analyses were performed using Python, release 3.9.6 (Python Software Foundation), with summary statistic generation using Pandas, version 1.3.3 (Python Software Foundation), interaction test using statsmodels, version 0.13.2 (Python Software Foundation), and other statistical tests using SciPy, version 1.8.0 (GitHub).

## Results

**Figure 1.**
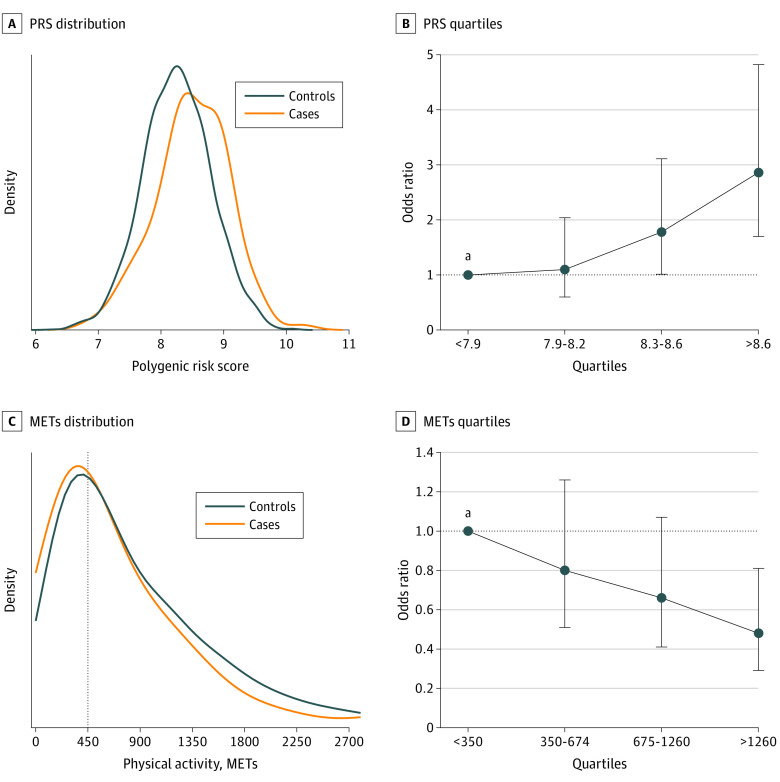
Association of Polygenic Risk Score (PRS) and Physical Activity Level With Risk for Gestational Diabetes A, The distributions of PRS between participants who developed gestational diabetes (cases) and those who did not (controls). B, Participants were divided into quartiles based on their PRS, with the odds ratio (OR) calculated against the reference group (lowest quartile). For quartile 2, the OR was 1.1 (95% CI, 0.6-2.0); for quartile 3, 1.8 (95% CI, 1.0-3.1); and for quartile 4, 2.9 (95% CI, 1.7-4.8). C, The distributions of physical activity levels measured in metabolic equivalents of task (METs) between gestational diabetes cases and controls. D, Participants were divided into 4 groups based on their METs, with the OR calculated against the reference group (lowest quartile). For quartile 2, the OR was 0.8 (95% CI, 0.5-1.3); for quartile 3, 0.7 (95% CI, 0.4-1.1); and for quartile 4, 0.5 (95% CI, 0.3-0.8). Kernel density plots in A and C were generated using the kdeplot function with default gaussian kernel. Each density was normalized independently with the argument common_norm set to false. ^a^Reference group (quartile 1).

Among the 3533 women included in this analysis (mean [SD] age, 28.6 [4.9] years), participants diagnosed with GD exhibited higher T2D PRS compared with controls (mean [SD] PRS, 8.49 [0.58] vs 8.25 [0.54]; *P* < .001, unpaired 2-tailed *t* test) (Figure 1A), with a prominent excess of cases in the highest PRS quartile. High PRS was associated with an increased risk of GD, particularly when the OR exceeded 2.5 (quartile 2: OR, 1.1 [95% CI, 0.6-2.0]; quartile 3: OR, 1.8 [95% CI, 1.0-3.1]; quartile 4: OR, 2.9 [95% CI, 1.7-4.8]) (Figure 1B). Higher levels of physical activity were associated with a reduced risk of GD (mean [SD] METs, 766.8 [754.8] vs 967.0 [962.1]; *P* = .003, unpaired 2-tailed *t* test) (Figure 1C). Participants diagnosed with GD exhibited significantly lower levels of physical activity in early pregnancy, compared with participants who were not diagnosed with GD, with those in the fourth quartile having ORs below 0.5 (quartile 2: OR, 0.8 [95% CI, 0.5-1.3]; quartile 3: OR, 0.7 [95% CI, 0.4-1.1]; quartile 4: OR, 0.5 [95% CI, 0.3-0.8]) (Figure 1D).

**Figure 2.**
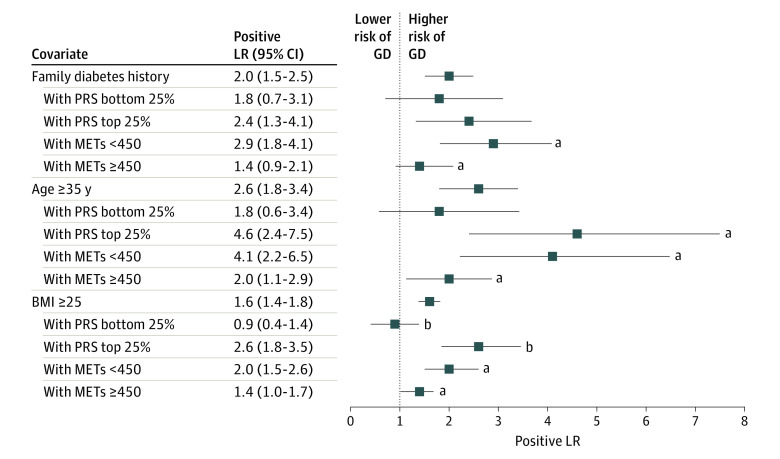
Positive Likelihood Ratio (LR) of Risk of Gestational Diabetes (GD) in the Context of Key Clinical Covariates Covariates include family history of diabetes, age 35 years or older, and body mass index (BMI; calculated as weight in kilograms divided by height in meters squared). Physical activity levels are measured in metabolic equivalents of task (METs). The positive LR values reflect the risk of developing GD among subgroup participants with the entire cohort used as the reference group. The positive LR *P* value against parent subgroup *P* value is the bootstrapped *P* value of the positive LR, where the reference group is the parent subgroup only. ^a^Bootstrapped *P* < .05. ^b^Bootstrapped *P* < .01.

We next established the association of PRS and METs with the risk of GD when participants were stratified based on 3 high-risk covariates: family history of diabetes, 35 years or older, and BMI of at least 25 (Figure 2 and Table 1). In high-risk population subgroups (BMI ≥25 or age ≥35 years), individuals with high PRS or low activity levels had increased odds of a GDM diagnosis of 25% to 75%. Participants with a family history of diabetes and high PRS, defined here as those with scores above the top 25th percentile, exhibited increased odds of a GD diagnosis compared with the remainder of participants (OR, 2.6 [95% CI, 1.5-4.5]). Similarly, a family history of diabetes with lower levels of physical activity (METs <450) was associated with increased odds for GD compared with the remainder of the cohort (OR, 3.3 [95% CI, 2.1-5.3]). Among the participants with family history of diabetes (positive LR, 2.0 [95% CI, 1.5-2.5]), those with high PRS had slightly increased odds of a GD diagnosis, but the result was not statistically significant (positive LR, 2.4 [95% CI, 1.3-3.7]; bootstrapped *P* = .20). On the other hand, those who also reported low levels of METs (<450) showed a larger and statistically significant increase in odds for GD diagnosis than the parent group (positive LR, 2.9 [95% CI, 1.8-4.1]; bootstrapped *P* = .02). A similar outcome was observed for the subgroups with low PRS, defined as those with scores below the 25th percentile, and those with higher levels of physical activity (METs ≥450). That is, among participants with family history of diabetes, low PRS was not associated with significantly lower odds for GD (positive LR, 1.8 [95% CI, 0.7-3.1]; bootstrapped *P* = .37), whereas high levels of physical activity were associated with a significant reduction in odds for GD among these participants (positive LR, 1.4 [95% CI, 0.9-2.1]; bootstrapped *P* = .01). This suggests that, for the patients with a family history of diabetes, information about physical activity may be more informative than the PRS. The METs threshold of 450 from the Nurses’ Health Study II^20^ was determined to be a well-selected value based upon our exploratory analysis (eFigure 2 in the Supplement).

**Table 1.** Association of PRS and METs With GD Risk in the Context of Key Clinical Covariates^a^

Covariate and subgroups	No. of participants		OR (95% CI)	*P* value^b^	Positive LR (95% CI)	*P* value^c^	*P* value^b^^,^^d^
	Cases	Controls					
Family history of diabetes	45	591	2.5 (1.7-3.6)	7.5 × 10^−6^	2.0 (1.5-2.5)	<.001	NA
With PRS bottom 25%	9	129	1.9 (0.9-3.7)	.10	1.8 (0.7-3.1)	.08	.37
With PRS top 25%	16	172	2.6 (1.5-4.5)	.002	2.4 (1.3-3.7)	.004	.20
With METs ≥450	21	377	1.5 (0.9-2.4)	.09	1.4 (0.9-2.1)	.07	.02
With METs <450	24	214	3.3 (2.1-5.3)	4.7 × 10^−6^	2.9 (1.8-4.1)	<.001	.01
Aged ≥35 y	33	332	3.1 (2.1-4.6)	1.1 × 10^−6^	2.6 (1.8-3.4)	<.001	NA
With PRS bottom 25%	6	88	1.8 (0.8-4.2)	.16	1.8 (0.6-3.4)	.14	.13
With PRS top 25%	14	78	5.1 (2.8-9.2)	5.4 × 10^−6^	4.6 (2.4-7.5)	<.001	.02
With METs ≥450	18	237	2.1 (1.3-3.5)	.009	2.0 (1.1-2.9)	.01	.03
With METs <450	15	95	4.5 (2.5-7.9)	9.9 × 10^−6^	4.1 (2.2-6.5)	<.001	.03
BMI ≥25	85	1368	2.7 (1.9-3.9)	6.2 × 10^−8^	1.6 (1.4-1.8)	<.001	NA
With PRS bottom 25%	12	347	0.9 (0.5-1.6)	.77	0.9 (0.4-1.4)	.32	.004
With PRS top 25%	34	337	3.2 (2.1-4.7)	3.2 × 10^−7^	2.6 (1.8-3.5)	<.001	.002
With METs ≥450	45	854	1.5 (1.1-2.2)	.03	1.4 (1.0-1.7)	.02	.05
With METs <450	40	514	2.4 (1.7-3.6)	1.4 × 10^−5^	2.0 (1.5-2.6)	<.001	.05

Abbreviations: BMI, body mass index (calculated as weight in kilograms divided by height in meters squared); GD, gestational diabetes; LR, likelihood ratio; METs, metabolic equivalents of task; NA, not applicable; OR, odds ratio; PRS, polygenic risk score.

^a^ The cases and controls include the number of participants in a subgroup on the left. The OR and positive LR values reflect the risk of developing GD among subgroup participants with the rest of the cohort used as the reference group for OR and the entire cohort for positive LR.

^b^ Calculated using the Fisher exact test.

^c^ Indicates bootstrapped *P* value of the positive LR, for which the reference group is all participants.

^d^ Positive LR *P* value against parent subgroup is the bootstrapped *P* value of the positive LR, for which the reference group is the parent subgroup only. For example, among participants with BMI of at least 25 (positive LR, 1.6), those with PRS in the bottom 25% have a significantly reduced GD risk (positive LR, 0.9), with bootstrapped *P* = .004. Furthermore, there is no statistical support that this subgroup (BMI ≥25 and PRS in the bottom 25%) differs from the entire cohort (*P* = .32).

In participants 35 years or older, both high PRS (OR, 5.1 [95% CI, 2.8-9.2]) and low METs (OR, 4.5 [95% CI, 2.5-7.9]) were associated with increased odds of a GD diagnosis compared with the rest of the cohort. When the analysis was restricted to the parent group of participants 35 years or older (positive LR, 2.6 [95% CI, 1.8-3.4]), we found that the subgroup with high PRS (positive LR, 4.6 [95% CI, 2.4-7.5]; bootstrapped *P* = .02) as well as the subgroup with low METs (positive LR, 4.1 [95% CI, 2.2-6.5]; bootstrapped *P* = .03) had further increased odds of a GD diagnosis. As with family history, the odds of a GD diagnosis were reduced for the participants with low PRS (positive LR, 1.8 [95% CI, 0.6-3.4]; bootstrapped *P* = .13) and high METs (positive LR, 2.0 [95% CI, 1.1-2.9]; bootstrapped *P* = .03).

In participants with BMI of at least 25, we similarly found that both high PRS (OR, 3.2 [95% CI, 2.1-4.7]) and low METs (OR, 2.4 [95% CI, 1.7-3.6]) reflect increased odds for GD compared with their respective remainders of the cohort. Among the participants with BMI of at least 25 (positive LR, 1.6 [95% CI, 1.4-1.8]), those with high PRS (positive LR, 2.6 [95% CI, 1.8-3.5]; bootstrapped *P* = .002) and those with low METs (positive LR, 2.0 [95% CI, 1.5-2.6]; *P* = .05) were both found to have increased odds of a GD diagnosis. The subgroups of participants with low PRS (positive LR, 0.9 [95% CI, 0.4-1.4]; bootstrapped *P* = .004) and those with high METs (positive LR, 1.4 [95% CI, 1.0-1.7]; *P* = .05) exhibited reduced odds of a GD diagnosis. Interestingly, the participants with BMI of at least 25 who had low PRS scores had baseline-level odds of a GD diagnosis (Figure 2).

**Figure 3.**
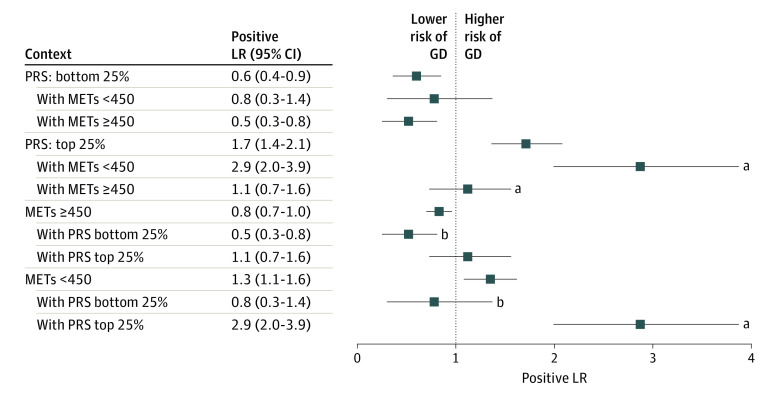
Positive Likelihood Ratio (LR) of Risk of Gestational Diabetes (GD) in the Context of the Cooperative Association of Polygenic Risk Score (PRS) and Physical Activity Levels Physical activity levels are measured in metabolic equivalents of task (METs). The positive LR values reflect the risk of developing GD among subgroup participants with the entire cohort used as the reference group. The positive LR *P* value against parent subgroup *P* value is the bootstrapped *P* value of the positive LR, where the reference group is the parent subgroup only. ^a^Bootstrapped *P* < .001. ^b^Bootstrapped *P* < .05.

Next, we examined the interaction between PRS and METs in association with the odds of developing GD (Figure 3 and Table 2). Participants with high PRS and low METs showed significantly higher odds of GD (OR, 3.4 [95% CI, 2.3-5.3]) in comparison with the remaining participants. Compared with participants with high PRS (positive LR, 1.7 [95% CI, 1.4-2.1]) or with low METs (positive LR, 1.3 [95% CI, 1.1-1.6]), those with both high PRS and low METs had increased odds of a GD diagnosis (positive LR, 2.9 [95% CI, 2.0-3.9]; bootstrapped *P* < .001). In contrast, participants with high PRS and high METs exhibited significantly lower positive LR compared with the parent subgroup of participants with high PRS (positive LR, 1.1 [95% CI, 0.7-1.6]; bootstrapped *P* < .001) and similar odds of a GD diagnosis to that at baseline (OR, 1.1 [95% CI, 0.7-1.8]; *P* = .55). Finally, participants with low PRS and high METs had significantly reduced risk of a GD diagnosis (OR, 0.5 [95% CI, 0.3-0.9]; *P* = .01) compared with participants outside of this group.

**Table 2.** Cooperative Association of PRS and METs With GD Risk^a^

Subgroup	No. of participants		OR (95% CI)	*P* value^b^	Positive LR (95% CI)	*P* value^c^	*P* value^d^
	Cases	Controls					
PRS bottom 25%	20	863	0.5 (0.3-0.9)	.007	0.6 (0.4-0.9)	.001	NA
With METs <450	8	264	0.8 (0.4-1.6)	.62	0.8 (0.3-1.4)	.21	.21
With METs ≥450	12	599	0.5 (0.3-0.9)	.01	0.5 (0.3-0.8)	.002	.21
PRS top 25%	55	829	2.2 (1.6-3.2)	2.1 × 10^−5^	1.7 (1.4-2.1)	<.001	NA
With METs <450	31	278	3.4 (2.3-5.3)	1.6 × 10^−7^	2.9 (2.0-3.9)	<.001	<.001
With METs ≥450	24	551	1.1 (0.7-1.8)	.55	1.1 (0.7-1.6)	.29	<.001
METs ≥450	74	2291	0.6 (0.4-0.9)	.008	0.8 (0.7-1.0)	.005	NA
With PRS bottom 25%	12	599	0.5 (0.3-0.9)	.01	0.5 (0.3-0.8)	.002	.016
With PRS top 25%	24	551	1.1 (0.7-1.8)	.55	1.1 (0.7-1.6)	.29	.062
METs <450	58	1110	1.6 (1.1-2.3)	.008	1.3 (1.1-1.6)	.005	NA
With PRS bottom 25%	8	264	0.8 (0.4-1.6)	.62	0.8 (0.3-1.4)	.21	.024
With PRS top 25%	31	278	3.4 (2.3-5.3)	1.6 × 10^−7^	2.9 (2.0-3.9)	<.001	<.001

Abbreviations: GD, gestational diabetes; LR, likelihood ratio; METs, metabolic equivalents of task; NA, not applicable; OR, odds ratio; PRS, polygenic risk score.

^a^ The cases and controls include the number of participants in a subgroup on the left. The OR and positive LR values reflect the risk of developing GD among subgroup participants with the rest of the cohort used as the reference group for OR and the entire cohort for positive LR.

^b^ Calculated using the Fisher exact test.

^c^ Indicates bootstrapped *P* value of the positive LR, for which the reference group is all participants.

^d^ Positive LR *P* value against parent subgroup is the bootstrapped *P* value of the positive LR, for which the reference group is the parent subgroup only. For example, among participants with METs of less than 450 (positive LR, 1.4), those with PRS in the top 25% have a significantly increased GD risk (positive LR, 2.9), with *P* < .001. Furthermore, this subgroup (METs <450 and PRS in the top 25%) also has significantly higher risk from the entire cohort (*P* < .001).

We performed a formal test for the interaction between PRS and METs using the logit model described in the Methods section. We estimate that, without confounding variables, *a* = −3.5, *d* = 0.42, *e* = 0.12, and *f* = 0.82, with the significance level for the interaction parameter *f* estimated as *P* = .03. In the presence of potential confounders (age and BMI), we estimate *a* = −8.5, *b* = 0.10, *c* = 0.08, *d* = 0.41, *e* = 0.10, and *f* = 0.78, with the significance level for the interaction parameter *f* estimated as *P* = .04 (eFigure 3 in the Supplement). We conclude that these data provide support for a nonadditive association between the 2 covariates.

Finally, we evaluated the ability of machine learning models to predict GD in the full cohort (baseline mean [SD] AUC, 0.710 [0.042]) using data from the first study visit only. The inclusion of PRS as a feature yielded modest improvement in predictive performance compared with the baseline GD prediction model (baseline plus PRS mean [SD] AUC, 0.734 [0.041]). The addition of METs led to a similarly modest increase in performance (baseline plus METs mean [SD] AUC, 0.708 [0.046]; baseline plus PRS plus METs mean [SD] AUC, 0.728 [0.045]).

## Discussion

We evaluated the association of 2 critical factors, genetic predisposition and physical activity, with the risk of GD among self-reported White nulliparous women with inferred European ancestry. We found that increased physical activity was associated with decreased risk of GD, and this reduction in risk was particularly significant in individuals who were genetically predisposed to diabetes through PRS or family history. These results suggest a nonadditive association between genetic predisposition and physical activity. Physically active participants were overall at lower risk for GD compared with other participants, regardless of PRS status. Similarly, participants with low PRS are at lower risk for GD compared with other participants, regardless of activity level. These findings are consistent with existing evidence on the importance of lifestyle factors in risk for GD^27^ and provide support for exercise interventions to improve pregnancy outcomes.^28^

Compared with previous studies on GD risk, this work focuses exclusively on nulliparous women. Although previous work^4,5^ has found that GD and other covariates from prior pregnancy are highly predictive of GD risk, this information was not available for our cohort. Further, the prediction of GD risk in first pregnancies has remained an ongoing challenge despite the broader availability of informative factors such as BMI, age, and family history. Previously, Kawai et al^14^ assessed the utility of PRS generated from loci associated with T2D in GD prediction and found a modest increase in predictive performance. Lamri et al^29^ reported similar conclusions in a South Asian birth cohort, highlighting the potential for increased prediction accuracy when both PRS and other known GD risk factors are considered. In the present study, we characterized the excess risk for GD that may be attributable to risk factors, including physical activity and PRS.

Although both high PRS and low physical activity levels have been shown to independently increase the risk for GD, the association between these 2 covariates is particularly valuable in clinical settings. Participants with high PRS and moderate to high activity levels in early pregnancy (METs ≥450) exhibit similar GD risk compared with the general population. Thus, increased physical activity recommendations for patients with a genetic predisposition may serve to ameliorate some excess GD risk. Further, these findings suggest the utility of PRS to stratify high-risk patients, which can be subject to targeted interventions to mitigate modifiable lifestyle risk factors at the appropriate stages of pregnancy.

### Strengths and Limitations

A strength of this work is the identification of the association between risk factors exhibited by patients who are most at risk for GD. Although the implementation of PRS in clinical practice needs expansion to more diverse populations and careful evaluation prospectively in intervention trials, these findings suggest that targeted interventions could reduce adverse perinatal outcomes among vulnerable patients. Furthermore, if patients with higher genetic risk constitute more clinically severe cases, this could allow for improved understanding of the pathophysiological pathways involved and better elucidate the mechanisms of GD risk.

This study has some limitations. First, the application of PRS with T2D markers was used for GD. Despite the reported similarities in genetic background between conditions, this may reduce the strength of our findings. The application of this PRS may suffer owing to significant variability in effect size for loci between the 2 conditions, as well as differences in the socioeconomic and genetic background of these patients in comparison to the genome-wide association study (GWAS) cohort used to derive the PRS.

Second, the PRS was calculated based on a GWAS consisting of European participants. Inclusion of SNVs from multiethnic GWAS or a GWAS from other population groups would demonstrate the effectiveness and generalizability of these metrics across other ethnic groups.

Third, the baseline features include quantitative and self-reported measurements that may have introduced uncertainty. Participants were selected for inclusion based on computed race and ethnicity. However, replication of these methods with either self-reported race or inferred ethnicity returns conclusions that are largely similar (eFigures 4-7 in the Supplement).

Finally, because many phenotypes have shown that relative risk varies with age, differences in age may account for differences in PRS performance when applied to cohorts of different demographic makeup.^30^ For diseases in which genetic relative risk decreases with age, genetic risk factors have stronger explanatory power among younger populations compared with older ones.

## Conclusions

The risk of a GD diagnosis increases significantly for individuals with high PRS as well as those with low levels of physical activity. Physical activity in early pregnancy is associated with reduced risk of GD and reversal of the excess risk in individuals with a strong genetic predisposition. Collectively, this work highlights the potential for targeted interventions to mitigate GD risk among nulliparous women who are at high risk through genetic predisposition, age, BMI, and family history of diabetes.

## Notes

Supplement. **eFigure 1.** Data Preprocessing Flowchart **eFigure 2.** Evaluation of METs Thresholds **eFigure 3.** Results of Statistical Analysis of Interaction Between PRS and METs **eFigure 4.** Influence of PRS and METs on the GD Risk in the Context of Key Clinical Covariates (Family Diabetes History, Age, and BMI) in Inferred European Participants **eFigure 5.** Cooperative Effects of PRS and METs on GD Risk in Inferred European Participants **eFigure 6.** Association of PRS and METs With the GD Risk in the Context of Key Clinical Covariates (Family Diabetes History, Age, and BMI) in Self-reported White Participants **eFigure 7.** Cooperative Effects of PRS and METs on GD risk in Self-reported White Participants
