# Supplementary material for: Association of Genetic Predisposition and Physical Activity With Risk of Gestational Diabetes in Nulliparous Women
Source: JAMA Netw Open. 2022 Aug 30;5(8):e2229158. doi: 10.1001/jamanetworkopen.2022.29158 (PMC9428742; doi:10.1001/jamanetworkopen.2022.29158)
Supplement: Supplement. — eFigure 1. Data Preprocessing Flowchart eFigure 2. Evaluation of METs Thresholds eFigure 3. Results of Statistical Analysis of Interaction Between PRS and METs eFigure 4. Influence of PRS and METs on the GD Risk in the Context of Key Clinical Covariates (Family Diabetes History, Age, and BMI) in Inferred European Participants eFigure 5. Cooperative Effects of PRS and METs on GD Risk in Inferred European Participants eFigure 6. Association of PRS and METs With the GD Risk in the Context of Key Clinical Covariates (Family Diabetes History, Age, and BMI) in Self-reported White Participants eFigure 7. Cooperative Effects of PRS and METs on GD risk in Self-reported White Participants [file jamanetwopen-e2229158-s001.pdf]

Supplementary Online Content

Pagel KA, Chu H, Ramola R, et al. Association of genetic predisposition and physical activity with risk of gestational diabetes in nulliparous women. *JAMA Netw Open*. 2022;5(8):e2229158. doi:10.1001/jamanetworkopen.2022.29158

eFigure 1. Data Preprocessing Flowchart

eFigure 2. Evaluation of METs Thresholds

eFigure 3. Results of Statistical Analysis of Interaction Between PRS and METs

eFigure 4. Influence of PRS and METs on the GD Risk in the Context of Key Clinical Covariates (Family Diabetes History, Age, and BMI) in Inferred European Participants

eFigure 5. Cooperative Effects of PRS and METs on GD Risk in Inferred European Participants

eFigure 6. Association of PRS and METs With the GD Risk in the Context of Key Clinical Covariates (Family Diabetes History, Age, and BMI) in Self-reported White Participants

eFigure 7. Cooperative Effects of PRS and METs on GD risk in Self-reported White Participants

This supplementary material has been provided by the authors to give readers additional information about their work.

© 2022 Pagel KA et al. *JAMA Network Open*.

eFigure 1. Data Preprocessing Flowchart

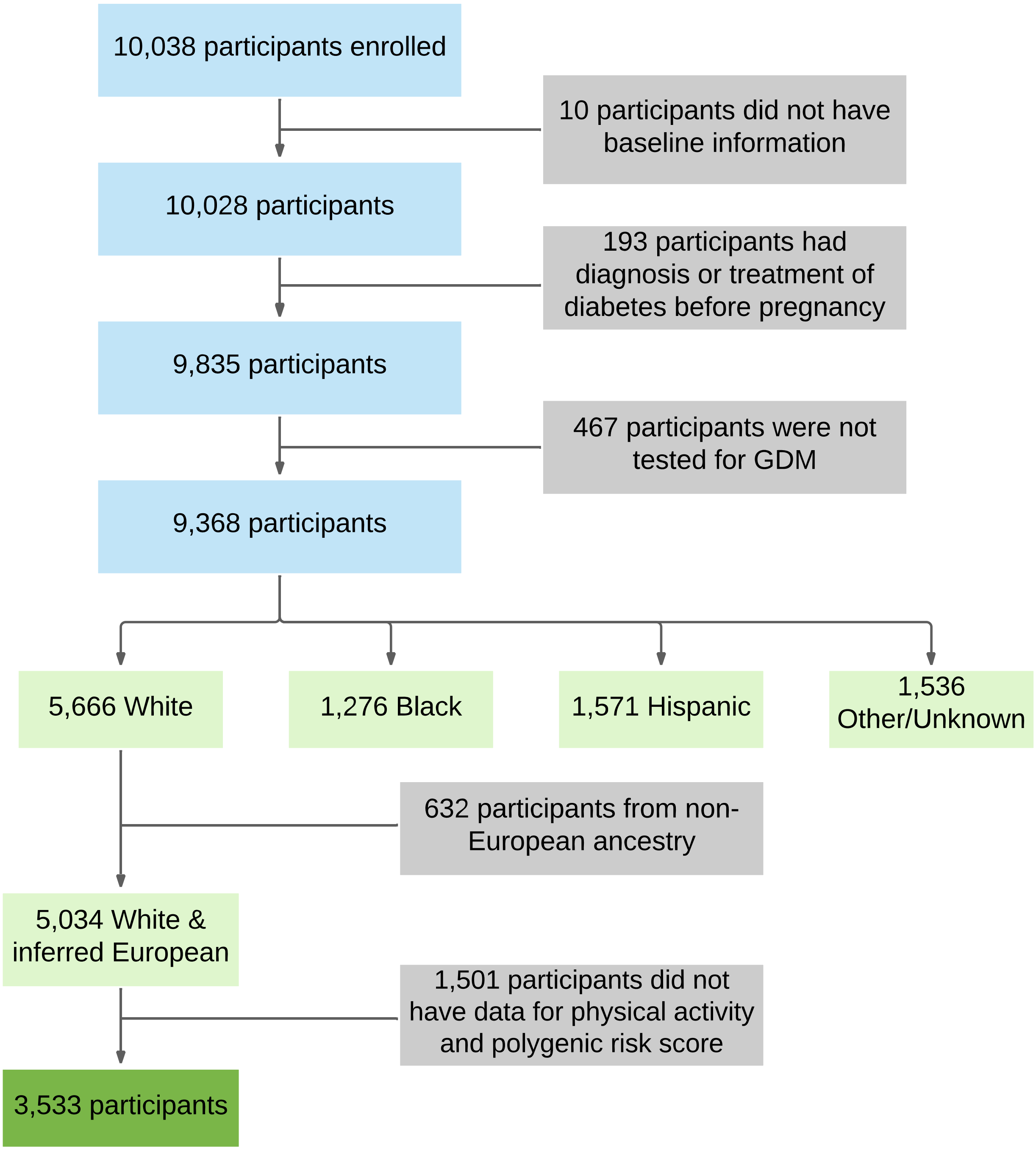

The filtering criteria used to generate the final cohort for analysis.

© 2022 Pagel KA et al. *JAMA Network Open*.

eFigure 2. Evaluation of METs Thresholds

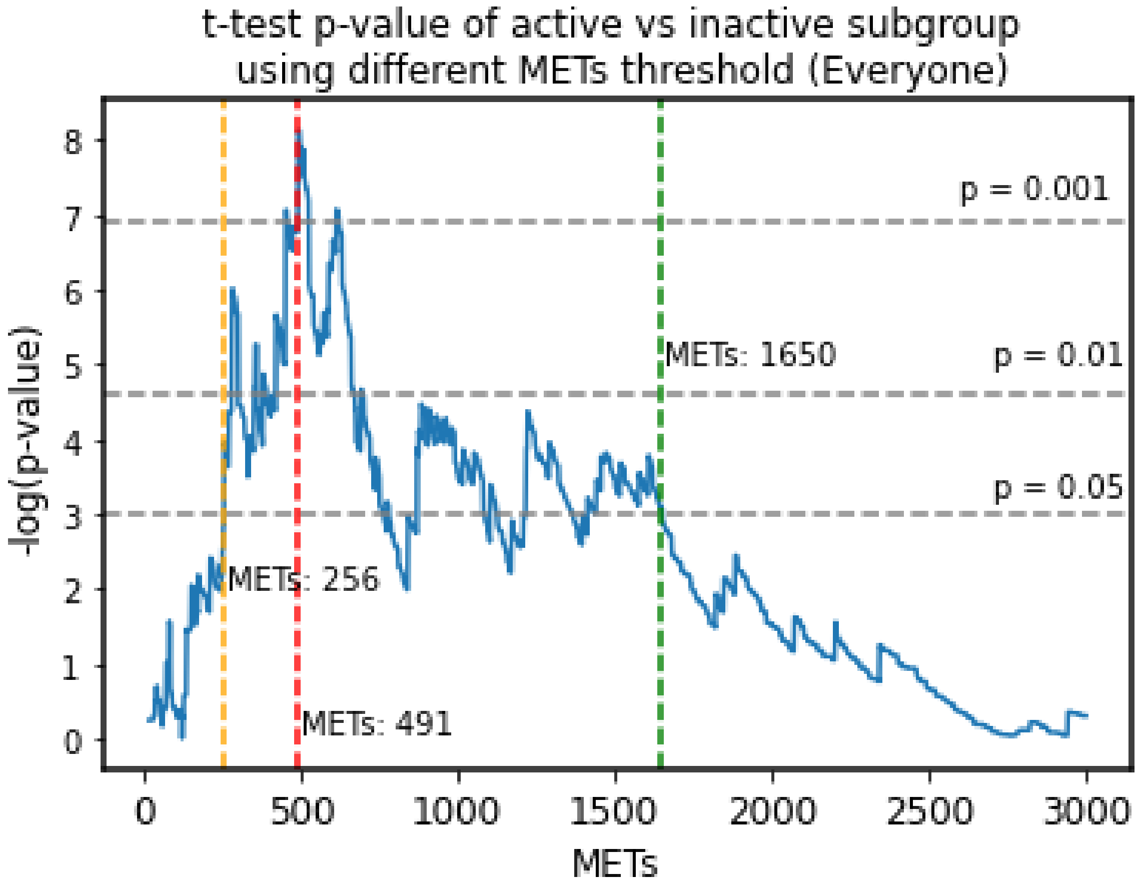

The p-value of GDM incidence for the METs-based subgroups. The cohort was split based on the METs threshold shown on the x-axis and the two-sample t-test p-value, shown on the y-axis, was calculated using the binary vectors of the incidence of GDM in each group (1 = cases, 0 = controls). The yellow (METs = 256) and green (METs = 1650) dashed lines show the lowest and the largest METs value with a p-value below 0.05. The red dashed line shows the METs value (METs = 491) with the strongest separation between the two groups, based on the p-value.

© 2022 Pagel KA et al. *JAMA Network Open*.

eFigure 3. Results of Statistical Analysis of Interaction Between PRS and METs

a)

Logit Regression Results

Dep. Variable:	GDM	No. Observations:	3533
Model:	Logit	Df Residuals:	3530
Method:	MLE	Df Model:	2
Date:	Sun, 05 Jun 2022	Pseudo R-squ.:	0.02193
Time:	10:29:18	Log-Likelihood:	-551.04
converged:	True	LL-Null:	-563.40
Covariance Type:	nonrobust	LLR p-value:	4.301e-06

	coef	std err	z	P>|z|	[0.025	0.975]
const	-3.6824	0.139	-26.537	0.000	-3.954	-3.410
high_prs	0.7851	0.181	4.331	0.000	0.430	1.140
inactive	0.4644	0.180	2.584	0.010	0.112	0.817

b)

Logit Regression Results

Dep. Variable:	GDM	No. Observations:	3533
Model:	Logit	Df Residuals:	3529
Method:	MLE	Df Model:	3
Date:	Sun, 05 Jun 2022	Pseudo R-squ.:	0.02631
Time:	10:29:19	Log-Likelihood:	-548.58
converged:	True	LL-Null:	-563.40
Covariance Type:	nonrobust	LLR p-value:	1.636e-06

	coef	std err	z	P>|z|	[0.025	0.975]
const	-3.5496	0.143	-24.747	0.000	-3.831	-3.268
high_prs	0.4159	0.253	1.643	0.100	-0.080	0.912
inactive	0.1216	0.243	0.502	0.616	-0.354	0.597
product	0.8184	0.372	2.202	0.028	0.090	1.547

c)

Logit Regression Results

Dep. Variable:	GDM	No. Observations:	3533
Model:	Logit	Df Residuals:	3528
Method:	MLE	Df Model:	4
Date:	Sun, 05 Jun 2022	Pseudo R-squ.:	0.08522
Time:	10:29:20	Log-Likelihood:	-515.39
converged:	True	LL-Null:	-563.40
Covariance Type:	nonrobust	LLR p-value:	6.894e-20

	coef	std err	z	P>|z|	[0.025	0.975]
const	-8.6272	0.673	-12.814	0.000	-9.947	-7.308
high_prs	0.7586	0.185	4.099	0.000	0.396	1.121
inactive	0.4286	0.188	2.284	0.022	0.061	0.797
Age_at_V1	0.0972	0.019	5.133	0.000	0.060	0.134
BMI	0.0774	0.012	6.543	0.000	0.054	0.101

d)

Logit Regression Results

Dep. Variable:	GDM	No. Observations:	3533
Model:	Logit	Df Residuals:	3527
Method:	MLE	Df Model:	5
Date:	Sun, 05 Jun 2022	Pseudo R-squ.:	0.08906
Time:	10:29:21	Log-Likelihood:	-513.22
converged:	True	LL-Null:	-563.40
Covariance Type:	nonrobust	LLR p-value:	4.448e-20

	coef	std err	z	P>|z|	[0.025	0.975]
const	-8.4780	0.674	-12.570	0.000	-9.800	-7.156
high_prs	0.4105	0.256	1.601	0.109	-0.092	0.913
inactive	0.1026	0.250	0.410	0.682	-0.387	0.593
product	0.7839	0.380	2.064	0.039	0.039	1.528
Age_at_V1	0.0965	0.019	5.093	0.000	0.059	0.134
BMI	0.0772	0.012	6.533	0.000	0.054	0.100

The components of the logit model using different sets of covariates as input and binary GDM status as output. high_prs: Binary encoding of whether an individual's PRS is at the highest quartile (Top 25%); inactive: Binary encoding of whether an individual's MET is below 450; product: the product of an individual's "high_prs" and "inactive" attribute; Age_at_V1: the age of the participant; BMI: the BMI of the participant. a) Logit model using only the "high_prs" and "inactive" as features. Both features are statistically significantly associated with GDM status ($p < 0.05$). b) Logit model using "high_prs", "inactive" and their product as features. The "high_prs" and "inactive" features are no longer statistically significant ($p > 0.05$) but their product is statistically significant. c) Logit model using "high_prs" and "inactive" as features, as well as two potential confounding variables age and BMI. Both "high_prs" and "inactive" are statistically significant after accounting for confounder variables. d) Logit model using "high_prs", "inactive" and their product as features, as well as potential confounding variables age and BMI. We observe a similar effect where "high_prs" and "inactive" are no longer statistically significant where the product remains statistically significant.

© 2022 Pagel KA et al. *JAMA Network Open*.

eFigure 4. Influence of PRS and METs on the GD Risk in the Context of Key Clinical Covariates (Family Diabetes History, Age, and BMI) in Inferred European Participants

Subgroup	Cases	Controls	OR	95% CI	P	LR⁺	95% CI	P	P*
Family DM History	47	631	2.3	(1.6, 3.3)	$1.91 \cdot 10^{-5}$	1.9	(1.4, 2.3)	<0.001	
+PRS Bottom 25%	10	138	1.9	(1.0, 3.7)	0.075	1.8	(0.8, 3.1)	0.083	0.420
+PRS Top 25%	16	182	2.4	(1.4, 4.1)	0.004	2.2	(1.2, 3.3)	0.008	0.223
+METs<450	25	229	3.1	(2.0, 4.9)	$7.66 \cdot 10^{-6}$	2.7	(1.8, 3.8)	<0.001	0.015
+METs≥450	22	402	1.4	(0.9, 2.3)	0.138	1.4	(0.9, 1.9)	0.087	0.015
Age>35	37	346	3.3	(2.2, 4.8)	$4.51 \cdot 10^{-8}$	2.7	(2.0, 3.5)	<0.001	
+PRS Bottom 25%	7	92	2.0	(0.9, 4.3)	0.103	1.9	(0.6, 3.5)	0.118	0.103
+PRS Top 25%	15	83	5.0	(2.8, 8.8)	$3.20 \cdot 10^{-6}$	4.5	(2.5, 7.2)	<0.001	0.025
+METs<450	17	101	4.7	(2.7, 8.0)	$1.62 \cdot 10^{-6}$	4.2	(2.4, 6.6)	<0.001	0.025
+METs≥450	20	245	2.2	(1.4, 3.6)	0.003	2.0	(1.3, 3.0)	0.003	0.025
BMI>25	93	1455	2.7	(1.9, 3.9)	$1.01 \cdot 10^{-8}$	1.6	(1.4, 1.8)	<0.001	
+PRS Bottom 25%	15	370	1.0	(0.6, 1.8)	0.889	1.0	(0.6, 1.5)	<0.001	0.009
+PRS Top 25%	37	353	3.2	(2.2, 4.7)	$7.34 \cdot 10^{-8}$	2.6	(1.9, 3.5)	<0.001	0.002
+METs<450	42	552	2.3	(1.6, 3.3)	$3.41 \cdot 10^{-5}$	1.9	(1.4, 2.4)	<0.001	0.079
+METs≥450	51	903	1.6	(1.2, 2.3)	0.006	1.4	(1.1, 1.8)	0.003	0.078

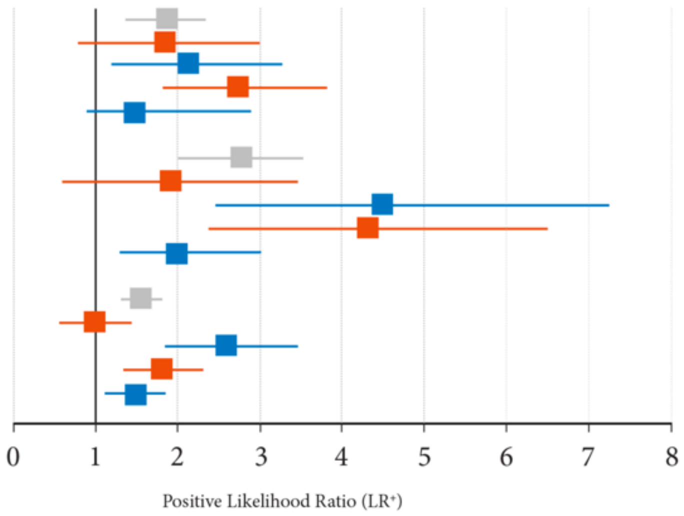

The cases and controls list the number of participants in a subgroup on the left. The OR and LR⁺ values reflect the risk of developing GDM among subgroup participants with the rest of the cohort used as the reference group for OR and the entire cohort for LR⁺. OR p-value (*P*) was determined using Fisher's exact test. LR⁺ p-value (*P*) is the bootstrapped p-value of the LR⁺, where the reference group is all participants. LR⁺ p-value against parent subgroup (*P**) is the bootstrapped p-value of the LR⁺, where the reference group is the parent subgroup only.

© 2022 Pagel KA et al. *JAMA Network Open*.

eFigure 5. Cooperative Effects of PRS and METs on GD Risk in Inferred European Participants

Subgroup	Cases	Controls	OR	95% CI	P	LR$^+$	95% CI	P	P*
PRS Bottom 25%	23	910	0.6	(0.4, 0.9)	0.010	0.6	(0.4, 0.9)	0.002	
+METs<450	9	287	0.8	(0.4, 1.5)	0.634	0.8	(0.3, 1.3)	0.186	0.216
+METs≥450	14	623	0.5	(0.3, 0.9)	0.017	0.6	(0.3, 0.8)	0.002	0.217
PRS Top 25%	60	874	2.2	(1.6, 3.2)	$7.02 \cdot 10^{-6}$	1.7	(1.4, 2.1)	<0.001	
+METs<450	33	297	3.3	(2.2, 5.0)	$1.29 \cdot 10^{-7}$	2.8	(2.0, 3.8)	<0.001	<0.001
+METs≥450	27	577	1.2	(0.8, 1.9)	0.356	1.2	(0.8, 1.6)	0.189	<0.001
METs≥450	82	2405	0.7	(0.5, 0.9)	0.018	0.9	(0.7, 1.0)	0.016	0.016
+PRS Bottom 25%	14	623	0.5	(0.3, 0.9)	0.017	0.6	(0.3, 0.8)	0.002	0.016
+PRS Top 25%	27	577	1.2	(0.8, 1.9)	0.356	1.2	(0.8, 1.6)	0.189	0.051
METs<450	61	1185	1.5	(1.1, 2.1)	0.018	1.3	(1.0, 1.5)	0.016	0.016
+PRS Bottom 25%	9	287	0.8	(0.4, 1.5)	0.634	0.8	(0.3, 1.3)	0.186	0.019
+PRS Top 25%	33	297	3.3	(2.2, 5.0)	$1.29 \cdot 10^{-7}$	2.8	(2.0, 3.8)	<0.001	<0.001

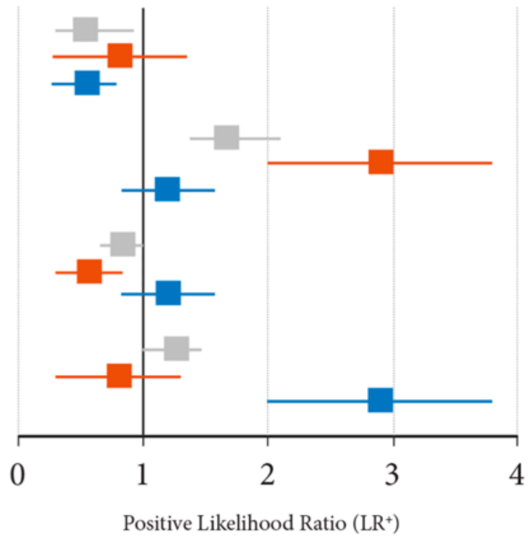

The cases and controls list the number of participants in a subgroup on the left. The OR and LR$^+$ values reflect the risk of developing GDM among subgroup participants with the rest of the cohort used as the reference group for OR and the entire cohort for LR$^+$. OR p-value (*P*) was determined using Fisher's exact test. LR$^+$ p-value (*P*) is the bootstrapped p-value of the LR$^+$, where the reference group is all participants. LR$^+$ p-value against parent subgroup (*P**) is the bootstrapped p-value of the LR$^+$, where the reference group is the parent subgroup only.

© 2022 Pagel KA et al. *JAMA Network Open*.

eFigure 6. Association of PRS and METs With the GD Risk in the Context of Key Clinical Covariates (Family Diabetes History, Age, and BMI) in Self-reported White Participants

Subgroup	Cases	Controls	OR	95% CI	P	LR^+	95% CI	P	P*
Family DM History	47	663	2.4	(1.7, 3.4)	$9.19 \cdot 10^{-6}$	1.9	(1.5, 2.4)	<0.001	
+PRS Bottom 25%	9	145	1.7	(0.9, 3.4)	0.122	1.7	(0.7, 2.9)	0.097	0.319
+PRS Top 25%	17	195	2.5	(1.5, 4.3)	0.002	2.3	(1.3, 3.5)	0.028	0.175
+METs<450	24	235	3.1	(2.0, 4.9)	$1.09 \cdot 10^{-5}$	2.7	(1.8, 3.9)	0.001	0.018
+METs≥450	23	428	1.5	(1.0, 2.4)	0.078	1.4	(0.9, 2.0)	0.054	0.018
Age>35	34	376	2.9	(1.9, 4.3)	$1.61 \cdot 10^{-6}$	2.4	(1.7, 3.2)	<0.001	
+PRS Bottom 25%	6	99	1.7	(0.7, 3.8)	0.276	1.6	(0.5, 3.2)	0.170	0.119
+PRS Top 25%	14	86	4.8	(2.6, 8.6)	$9.47 \cdot 10^{-6}$	4.4	(2.3, 7.1)	<0.001	0.017
+METs<450	15	104	4.2	(2.4, 7.5)	$1.66 \cdot 10^{-5}$	3.9	(2.1, 6.2)	<0.001	0.033
+METs≥450	19	272	2.0	(1.2, 3.3)	0.008	1.9	(1.1, 2.8)	0.012	0.033
BMI>25	91	1497	2.8	(2.0, 4.0)	$3.58 \cdot 10^{-9}$	1.6	(1.4, 1.8)	<0.001	
+PRS Bottom 25%	13	377	0.9	(0.5, 1.6)	0.886	0.9	(0.5, 1.5)	0.379	0.004
+PRS Top 25%	36	371	3.2	(2.1, 4.7)	$1.27 \cdot 10^{-7}$	2.6	(1.9, 3.4)	<0.001	0.002
+METs<450	42	551	2.5	(1.7, 3.6)	$5.70 \cdot 10^{-6}$	2.0	(1.5, 2.6)	<0.001	0.043
+METs≥450	49	946	1.6	(1.1, 2.3)	0.010	1.4	(1.1, 1.7)	0.007	0.043

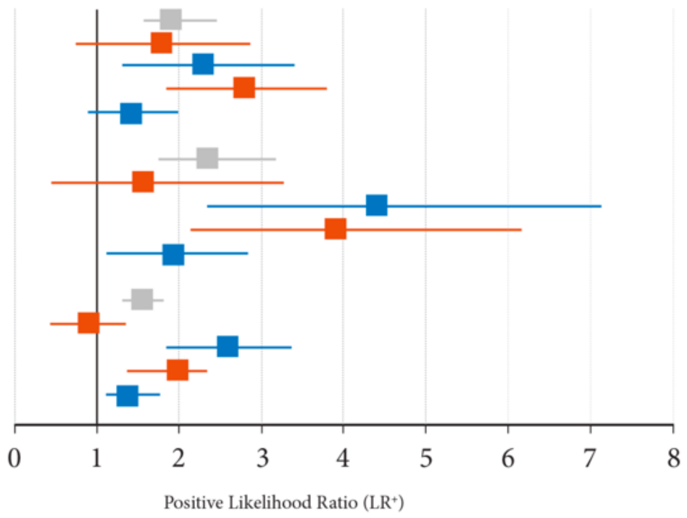

The cases and controls list the number of participants in a subgroup on the left. The OR and LR^+ values reflect the risk of developing GDM among subgroup participants with the rest of the cohort used as the reference group for OR and the entire cohort for LR^+. OR p-value (*P*) was determined using Fisher's exact test. LR^+ p-value (*P*) is the bootstrapped p-value of the LR^+, where the reference group is all participants. LR^+ p-value against parent subgroup (*P**) is the bootstrapped p-value of the LR^+, where the reference group is the parent subgroup only.

© 2022 Pagel KA et al. *JAMA Network Open*.

eFigure 7. Cooperative Effects of PRS and METs on GD risk in Self-reported White Participants

Subgroup	Cases	Controls	OR	95% CI	P	LR⁺	95% CI	P	P*
PRS Bottom 25%	21	948	0.5	(0.3, 0.8)	0.005	0.6	(0.4, 0.8)	0.001	
+METs<450	9	288	0.8	(0.4, 1.6)	0.744	0.8	(0.4, 1.5)	0.286	0.129
+METs≥450	12	660	0.4	(0.2, 0.8)	0.004	0.5	(0.2, 0.8)	<0.001	0.129
PRS Top 25%	57	913	2.2	(1.5, 3.0)	$2.39 \cdot 10^{-5}$	1.7	(1.3, 2.0)	<0.001	
+METs<450	32	302	3.4	(2.3, 5.1)	$1.20 \cdot 10^{-7}$	2.8	(2.0, 3.8)	<0.001	<0.001
+METs≥450	25	611	1.1	(0.7, 1.7)	0.640	1.1	(0.7, 1.5)	0.313	<0.001
METs≥450	79	2542	0.6	(0.4, 0.9)	0.007	0.8	(0.7, 1.0)	0.005	
+PRS Bottom 25%	12	660	0.4	(0.2, 0.8)	0.004	0.5	(0.2, 0.8)	<0.001	0.007
+PRS Top 25%	25	611	1.1	(0.7, 1.7)	0.640	1.1	(0.7, 1.5)	0.313	0.073
METs<450	60	1195	1.6	(1.1, 2.3)	0.007	1.3	(1.1, 1.6)	0.005	
+PRS Bottom 25%	9	288	0.8	(0.4, 1.6)	0.744	0.8	(0.4, 1.5)	0.281	0.039
+PRS Top 25%	32	302	3.4	(2.3, 5.1)	$1.20 \cdot 10^{-7}$	2.8	(2.0, 3.8)	<0.001	<0.001

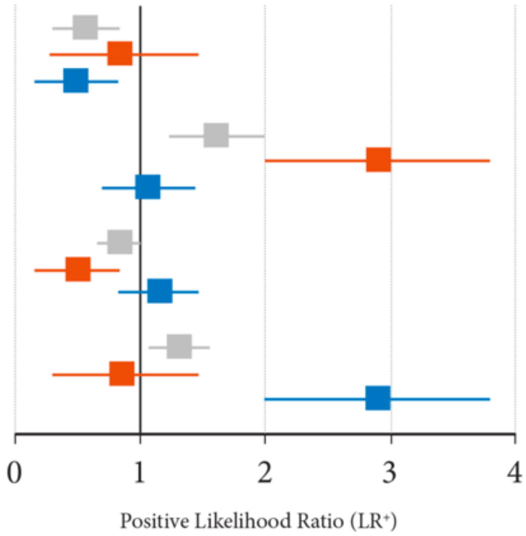

The cases and controls list the number of participants in a subgroup on the left. The OR and LR⁺ values reflect the risk of developing GDM among subgroup participants with the rest of the cohort used as the reference group for OR and the entire cohort for LR⁺. OR p-value (*P*) was determined using Fisher's exact test. LR⁺ p-value (*P*) is the bootstrapped p-value of the LR⁺, where the reference group is all participants. LR⁺ p-value against parent subgroup (*P**) is the bootstrapped p-value of the LR⁺, where the reference group is the parent subgroup only.

© 2022 Pagel KA et al. *JAMA Network Open*.
